# Gene Expression and DNA Methylation in Human Papillomavirus Positive and Negative Head and Neck Squamous Cell Carcinomas

**DOI:** 10.3390/ijms231810967

**Published:** 2022-09-19

**Authors:** Snežana Hinić, April Rich, Nicole V. Anayannis, Stephanie Cabarcas-Petroski, Laura Schramm, Patricio I. Meneses

**Affiliations:** 1 Department of Biological Sciences, Fordham University, Bronx, NY 10458, USA; 2 Department of Human Genetics, Radboud Institute for Molecular Life Sciences, Radboud University Medical Center, 6525GA Nijmegen, The Netherlands; 3 Department of Computational and Systems Biology, University of Pittsburgh, Pittsburgh, PA 15213, USA; 4 Biology Department, Pennsylvania State University, Beaver Campus, Monaca, PA 15061, USA; 5 Department of Biological Sciences, St. John’s University, Queens, NY 11439, USA

**Keywords:** cancer, head and neck cancer, gene expression, methylation, HPV

## Abstract

High-risk human papillomaviruses (HPV) are important agents, responsible for a large percentage of the 745,000 cases of head and neck squamous cell carcinomas (HNSCC), which were identified worldwide in 2020. In addition to being virally induced, tobacco and heavy alcohol consumption are believed to cause DNA damage contributing to the high number of HNSCC cases. Gene expression and DNA methylation differ between HNSCC based on HPV status. We used publicly available gene expression and DNA methylation profiles from the Cancer Genome Atlas and compared HPV positive and HPV negative HNSCC groups. We used differential gene expression analysis, differential methylation analysis, and a combination of these two analyses to identify the differences. Differential expression analysis identified 1854 differentially expressed genes, including *PCNA*, *TNFRSF14*, *TRAF1*, *TRAF2*, *BCL2*, and *BIRC3*. *SYCP2* was identified as one of the top deregulated genes in the differential methylation analysis and in the combined differential expression and methylation analyses. Additionally, pathway and ontology analyses identified the extracellular matrix and receptor interaction pathway as the most altered between HPV negative and HPV positive HNSCC groups. Combining gene expression and DNA methylation can help in elucidating the genes involved in HPV positive HNSCC tumorigenesis, such as *SYCP2* and *TAF7L*.

## 1. Background

Head and neck squamous cell carcinomas (HNSCC) are a group of cancers from anatomically distinct areas: Oropharynx, larynx, hypopharynx, oral cavity, and tongue. HNSCC accounted for approximately 745,000 cancer cases worldwide in 2020, which is an alarming number [1,2]. Etiologic agents identified as causes of HNSCC include alcohol consumption and tobacco use, and high-risk human papillomavirus (HPV) infection [3,4]. Changes in sexual behavior have been found to be associated with higher HPV oral and oropharyngeal incidence and HPV is becoming increasingly indicated as one of the major HNSCC etiologic agents [5,6,7,8,9]. The predominant HPV genotype identified in HNSCC is HPV 16 (in as many as 90% of cases).

HPVs are non-enveloped, double-stranded DNA viruses with a genome that includes six early expressed genes, and two late expressed genes [10]. Two of the early genes, *E6* and *E7*, are characterized as oncogenes in cervical, oral, anal, and penile cases [11,12,13]. *E6* interferes with cell survival pathways by targeting p53 for proteasome degradation, and *E7* promotes cell proliferation interfering with the function of the Retinoblastoma protein (pRb) [11,12].

Genes involved in cancer development and progression can affect cell proliferation, metastasis, and invasion [14]. As a result of HPV infection, pathways that control cytoskeletal rearrangement, immune response, extracellular matrix formation, and receptor activation are differentially altered [14,15,16]. These genetic changes sustained during carcinogenesis and viral oncogenesis are a result of changes in gene expression and transcriptome profile. HNSCC onset, progression, and outcome differ depending on the presence or absence of HPV. In HPV negative (HPVN) HNSCC patients, the tumor suppressor genes *TP53* and *p16*, along with *CCND1* oncogene are the most frequently identified mutated genes [17,18]. In HPV positive (HPVP) HNSCC patients, the viral oncogenes *E6* and *E7* initiate deregulation by targeting p53 and pRb, respectively [11,12]. Studies have started to describe epigenetic profile changes, specifically on the level of DNA methylation, and it has been reported that the methylation status in HNSCC patients is associated with HPV infection (i.e., positive versus negative) [19,20,21,22].

Recently, there has been a growing interest and need for understanding the biological significance of HPVP and HPVN HNSCC. Many of these studies have utilized tools of the rapidly expanding field of bioinformatics [23,24,25].

We performed a meta-analysis of The Cancer Genome Atlas (TCGA) HPVP and HPVN HNSCC transcriptome and DNA methylome data [26,27]. To our knowledge, this is the first time a study bridges these two datasets and compares groups based on the HPV status in HNSCC patient samples. Our findings show that pathways involved in viral invasion and trafficking, as well as immune system activation are differentially expressed in HPVP HNSCC. We identified that the differential expression of these pathways positively correlates with the differential methylation analysis.

This study demonstrates the ability of computational methods to identify biomarkers of potential clinical significance from a centralized resource of available datasets, such as TCGA.

## 2. Results

### 2.1. TCGA HNSCC HPVP and HPVN Patients Have Comparable Clinical History

**Figure 1:**
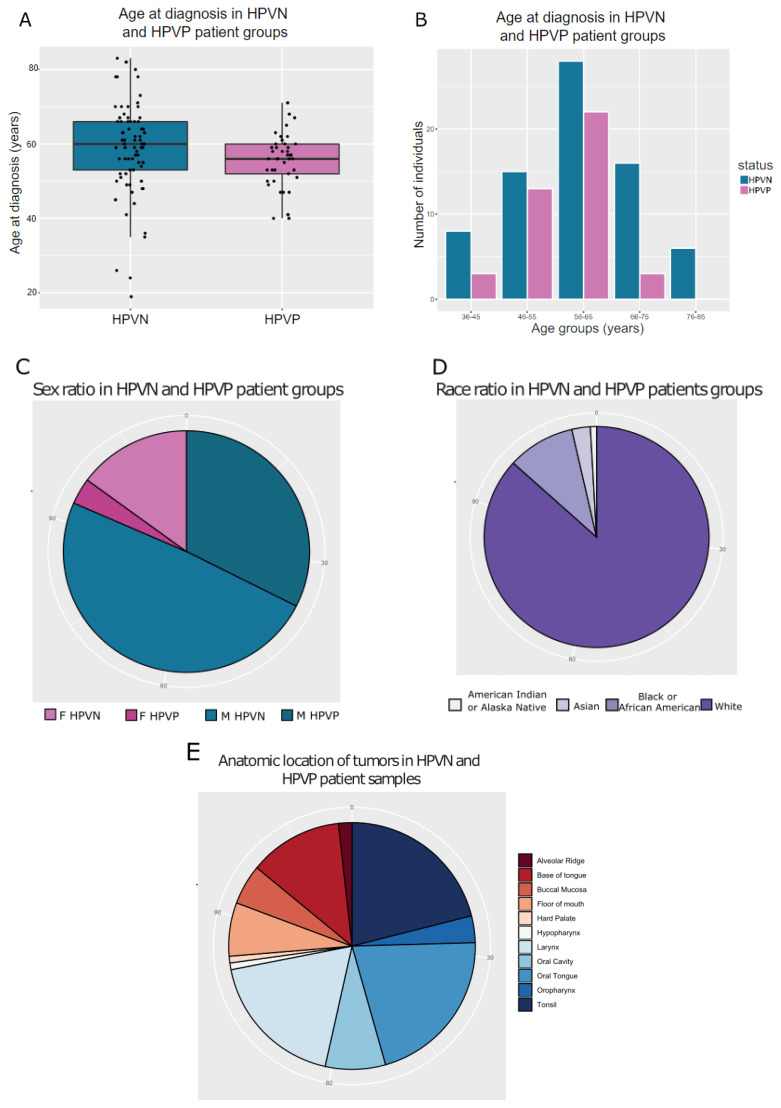

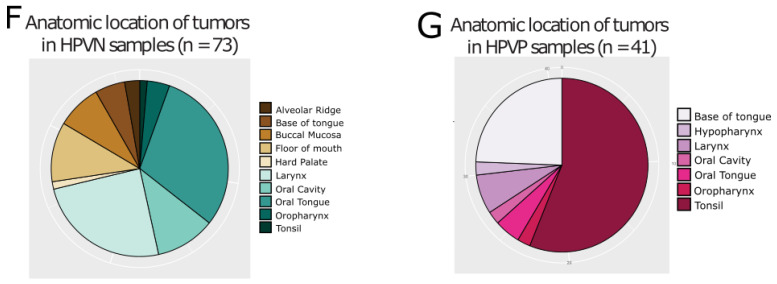
Clinical data of the TCGA HNSCC HPV- positive and negative patients. Patients were filtered according to the HPV status using the information regarding *p16* expression and in situ hybridization information (only patients with information present were included in the study; *n* = 114, HPVP = 41, HPVN = 73). (**A**) Distribution of age at cancer diagnosis between two groups of patients, HPVP and HPVP; (**B**) distribution of patients in different age groups, and sidewise comparison of age groups and HPV status; (**C**) representation of male and female patients, HPVP and HPVN; (**D**) representation of different races, independent of sex or HPV status; (**E**) distribution of different anatomical sites where cancer originated; (**F**,**G**) a closer look at the specific location of HPVN patients (**F**) and HPVP (**G**).

To ensure that the data from the TCGA database were comparable, we first examined the clinical profile of the patients in both HPVP and HPVN patient groups. Patient age distribution showed that the median values were comparable in both HPVN and HPVP groups (HPVN = 59, HPVP = 58 years) (Figure 1A). Most patients were grouped into 56–65 years of age range, and the collective (both for HPVP and HPVN groups) median age was in the same age range, as well (median = 58.5 years) (Figure 1B). We observed that there were no HPVP patients in the oldest patient category of 76–85 years of age (Figure 1B). Sex distribution in our sample groups revealed that females (*n* = 21) were underrepresented in comparison to males (*n* = 93) (Figure 1C). According to TCGA’s classification in different race categories, race distribution showed that the white race was significantly the most represented one (*n* = 98) (Figure 1D). Anatomical site analysis of these HNSCC showed that there were apparent differences in the location of tumor depending on the HPV status (Figure 1E–G). HPVP cancers were primarily found in the tonsil region and the base of the tongue, and HPVN cancers were primarily in the oral tongue and the larynx (Figure 1F,G).

### 2.2. Clustering of Samples Confirms That HPVN and HPVP Are Two Separate Comparable Groups

**Figure 2:**
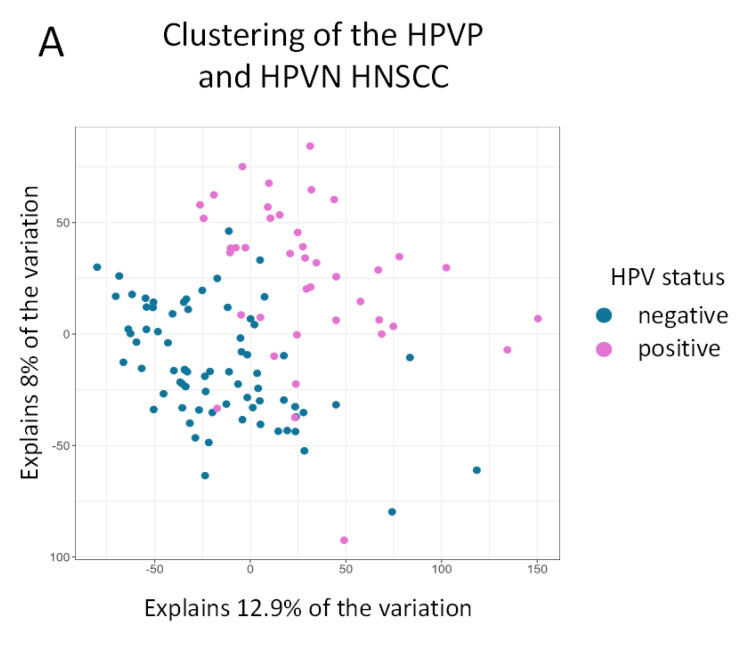

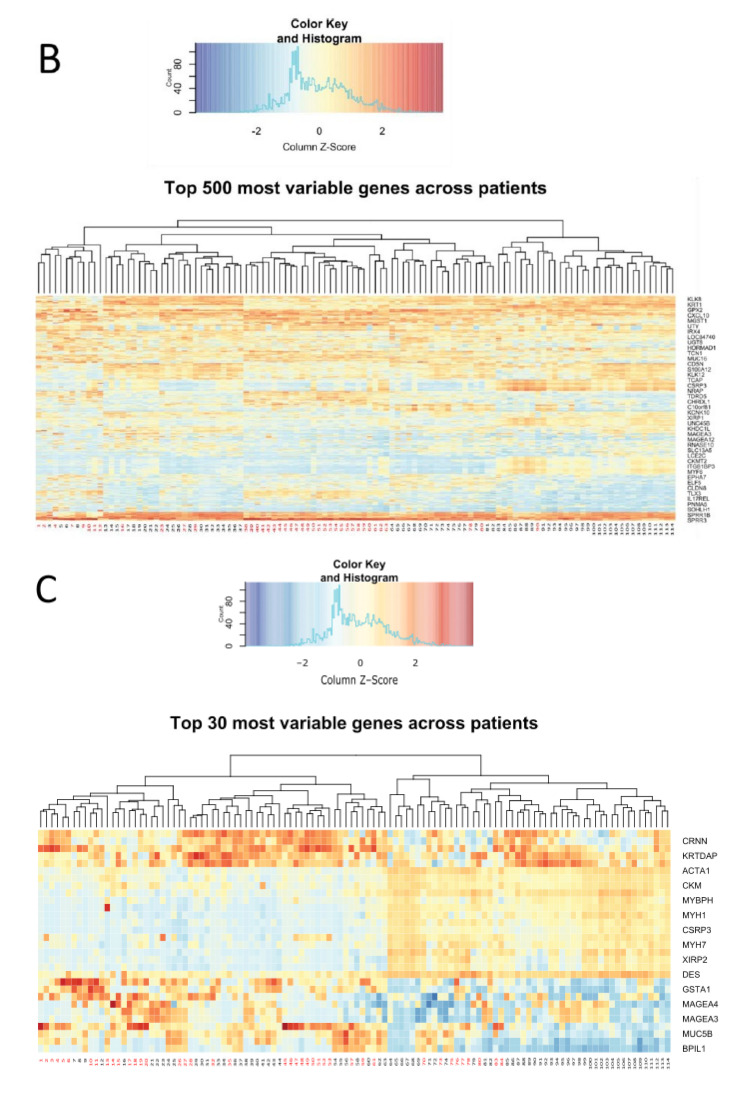

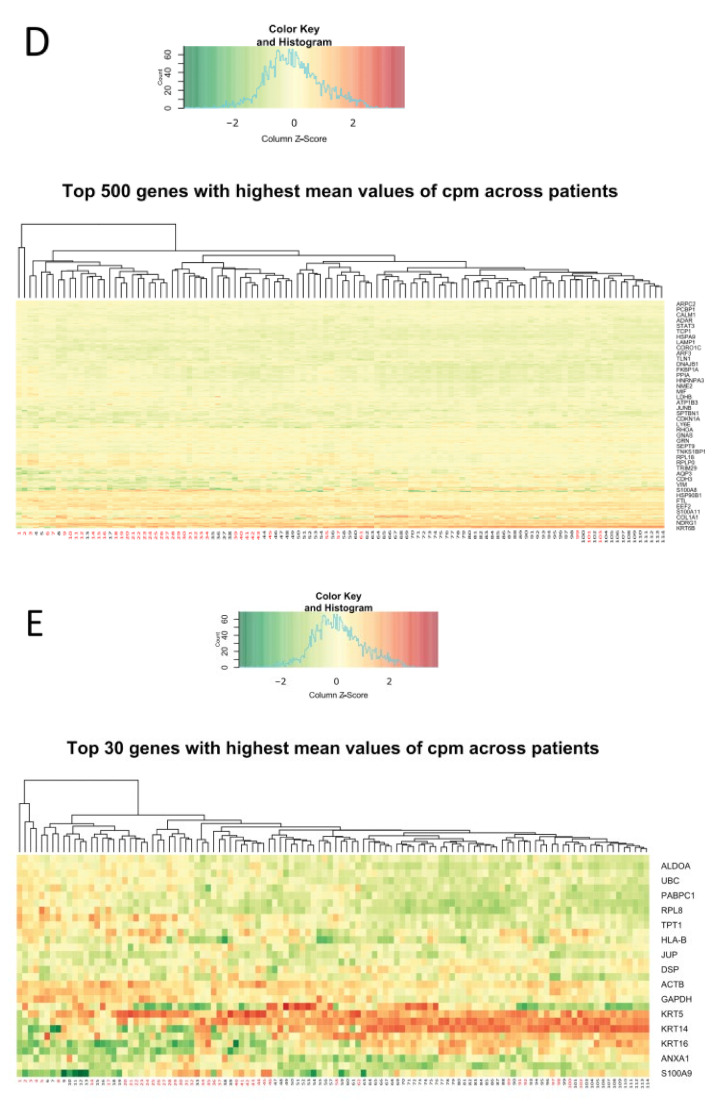
Clustering of the TCGA HNSCC HPVP and HPVN samples and genes. PCA and heatmap clustering shows distinct patient groups as we classify them. (**A**) PCA shows that patients classify in two separate groups for the most part, confirming that separation in HPVP and HPVN groups by *p16* expression and in situ hybridization was a valid parameter; (**B**–**E**) are heatmaps of the most variable genes (**B**,**C**) and most abundant transcripts (**D**,**E**) among *n* = 114 HNSCC samples; (**B**) shows the top 500 most variable genes, while a closer look at the top 30 most variable genes is shown in (**C**); top 500 transcripts with the highest mean values are depicted in (**D**) with a zoomed-in perspective to the top 30 in (**E**) HPVN samples (labeled in black) and HPVP (in red colored numbers).

To explore clustering and similarity of samples, we analyzed the two experimental groups, HPVN and HPVP, by PCA and clustering on heatmap. PCA revealed that the two groups (detailed explanation in Material and Methods) clustered mostly as two separate and distinct groups with an overlapping middle area (Figure 2A, HPVN in blue, HPVP in pink).

**Figure 3:**
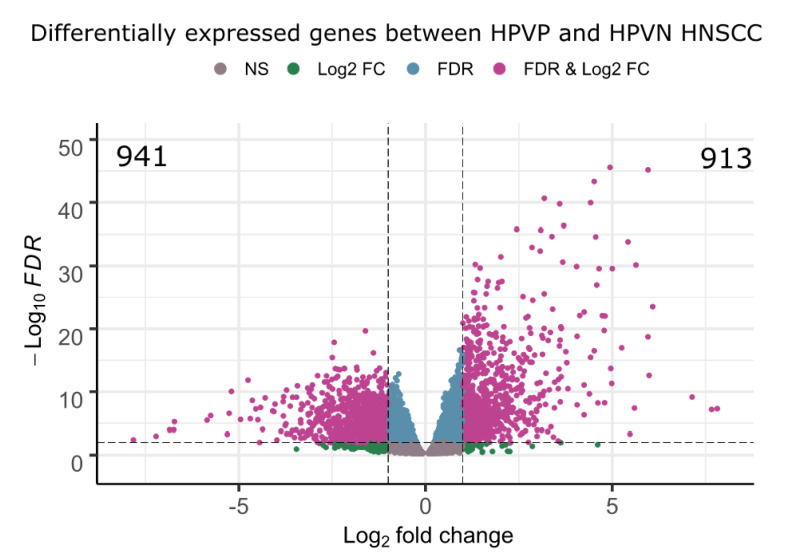
Volcano plot depicting differentially expressed genes. Differential expression analysis identified 1854 DEGs, 941 were downregulated and 913 were upregulated between HPVP and HPVN HNSCC patient groups (using HPVN as a baseline for comparison). Purple represents DEGs, blue is statistically significant according to the *p*-value, green is statistically significant according to the logFC, while grey is not statistically significant.

Heatmaps (Figure 2B–E) revealed specific patterns:

1. The pattern of specific groups of genes in a larger scale analysis of the top 500 most variable genes (Figure 2B) and of the top 30 most variable genes (Figure 2C), remained consistent. Genes including kallikreins family genes (serine proteases) remained highly variable between the patients [28,29]. Keratin, a structural component was found as variable, as well as oxidative damage protection proteins (GPX2), cytokines, inflammatory response genes, immune response genes, and cell cycle controlling genes. Figure 2C depicts a gene responsible for stratification of the skin (*KRTDAP*), that was highly variably expressed, as well as an epithelial immune response and differentiation gene (*CRNN*). At a larger scale, genes from two groups of patients seemed to cluster mostly separately, significantly resembling the clustering observed in PCA (Figure 2A), with an intermediate overlapping cluster of samples (Figure 2B).
2. Top 500 and top 30 most abundant transcripts clustered mostly in two different groups (Figure 2D and Figure 3E, respectively). Notably, some of the genes with the highest numbers of transcripts were cell cycle checkpoint genes, cytoskeletal regulatory genes, and immune response genes.

### 2.3. Transcriptome Analysis Identified 1854 Differentially Expressed Genes among HPVN and HPVP HNSCC Groups

To explore the impact of HPV on gene expression in HNSCC, we performed differential gene expression analysis (DGE) using TCGAbiolinks Bioconductor package for R (Material and Methods). Using FDR ≤ 0.01 and │logFC│ ≥ 1, with HPVN as baseline, DGE identified 1854 differentially expressed genes (DEG), 941 downregulated and 913 upregulated in HPVP samples (Figure 3). Significant DEGs are in purple, and genes that are non-significant or significant by one of the parameters are in grey, blue, and green. Some of the key representative DEGs are: *PCNA*, *TNFRSF14*, *TRAF1*, *TRAF2*, *BCL2*, and *BIRC3*.

To functionally explain the up- and downregulated genes, we performed KEGG analysis [30]. Table 1 presents ten of the significantly enriched pathways (a full list of DEGs and KEGG pathways can be found in Supplementary Tables S1 and S3, respectively) [30,31]. The KEGG enriched pathways included those involved in ECM-interaction, cytokine production, cell cycle regulators, apoptosis, and genes identified as part of an HPV infection.

Pathway and ontology analyses were performed using the Enrichr and PANTHER classification systems (Table 2 and Table 3) [32,33,34]. These tools identified similar pathways and patterns as KEGG (Table 2, Table 3 and Table 4, Supplementary Table S2). The top KEGG significantly enriched pathways (i.e., enrichment of genes) were consistent with HPV infection (Table 4). Notably, transcription factors and genes involved in cell cycle progression were identified as upregulated. In contrast, genes involved in cellular response to stimulus, including chemotherapeutic agents and radiation were downregulated [35].

### 2.4. DNA Methylome Analysis Showed HPVP and HPVN HNSCC Methylation Levels Were Comparable

**Figure 4:**
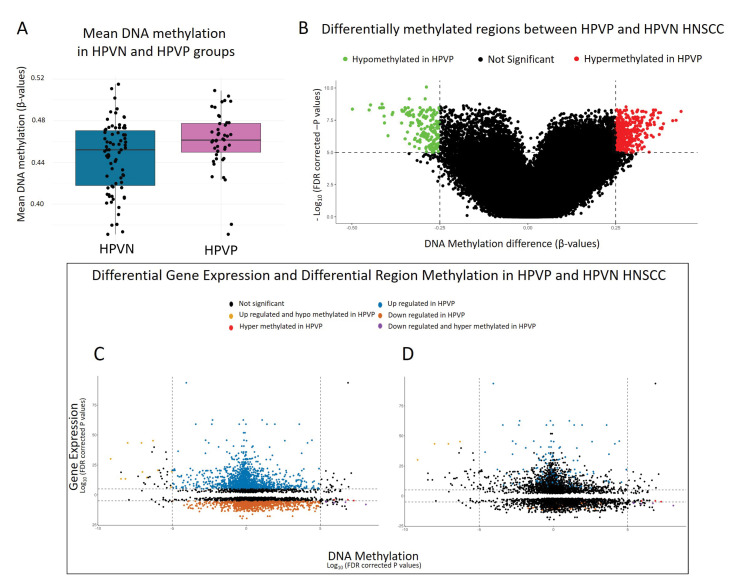
Differential methylation in HNSCC patients. Represented above is the methylation profile in HNSCC. (**A**) Mean methylation between HPVN and HPVP HNSCC patient samples; (**B**) volcano plot showing the hypomethylated genes in green and hypermethylated genes in red. HPVN samples are used as a baseline. We used $\underline{\beta \;}$ ≥ 0.25 and *p* ≤ 10^−5^; (**C**,**D**) show a Starburst plot that combined differential gene expression data with differential methylation data. HPVN is used as a baseline. We used $\underline{\beta \;}$ ≥ 0.25, FDR_expression_ ≤ 10^−5^, FDR_DNAmethylation_ ≤ 10^−5^ │logFC│ ≥ 1 in (**C**) and more stringent parameters $\underline{\beta \;}$ ≥ 0.25, FDR_expression_ ≤ 10^−5^, FDR_DNAmethylation_ ≤ 10^−5^ │logFC│ ≥ 3 in (**D**).

To explore epigenetic changes in HNSCC due to HPV, we focused on DNA methylation. We performed a differential methylation analysis (DMA) using the following parameters: $\bar{\beta \;}$ ≥ 0.25 and *p* ≤ 10^−5^ that identified top hypo- and hypermethylated regions of the genome and genes involved (Table 5, Supplementary Table S3). We compared the overall median methylation levels of our two groups of patient samples, HPVN and HPVP, and observed that their median values were comparable ($\bar{\beta \;}$~0.46) (Figure 4A). DMA results are represented on a volcano plot comparing hypomethylated and hypermethylated regions in HPVP (HPVN samples used as baseline comparison) (Figure 4B).

### 2.5. Starburst: An Analysis That Bridges Differentially Expressed and Methylated Genes Revealed Similar Patterns to DNA Methylome Analysis and Potential Biomarker Gene for HPVP HNSCC

To identify common DEG and DMA genes, we performed a Starburst analysis [36]. This analysis identifies genes with similar DEG and DMR patterns (i.e., hypomethylated and upregulated and hypermethylated and downregulated), using the following parameters: $\bar{\beta \;}$ ≥ 0.25, *FDR_expression_* ≤ 10^−5^, *FDR_DNAmethylation_* ≤ 10^−5^. Our analysis showed that a similar pattern was observed with │logFC│, which is set to ≥ 1, and more stringent │logFC│ set to be ≥ 3. The pattern of DEG and DMR expression remained comparable with both parameters used, and the top statistically significant DEG and DMR identified in both analyses were consistent (Figure 4C,D). We decided to proceed with │logFC│ ≥ 1 and depict some of the representative results (Table 6), and a complete list can be found in Supplementary Table S5.

## 3. Discussion

HPV has been recognized as an important driver of HNSCC [23,37,38]. The patient treatment varies depending on HPV positive (HPVP) versus negative (HPVN) HNSCC; therefore, it is important to gain further knowledge of the genetic profile of HNSCC. Our study showed that HPVP HNSCC patients exhibit gene deregulation at gene transcription and methylation levels different from HPVN HNSCC patients. When analyzed, both independently and collectively, gene expression and methylation deregulation patterns specifically point out changes in gene pathways including those involved in controlling invasion, immune response, differentiation, and cell division.

In total, the cohort of patient’s samples analyzed was 114 (HPVN = 73 and HPVP = 41). There was a disparity in the male/female self-described sample ratio, where male samples accounted for 93, and female samples the remaining 21 (Figure 1C). A possible explanation for this disparity might be that HNSCC cases are sex biased and more prevalent in males, but a larger cohort needs to be analyzed to address this disparity. Moreover, there was an overrepresentation of white race (*n* = 98) in this cohort for HNSCC (Figure 1D). This lack of racial representation is unfortunately not uncommon in clinical studies. We have since identified studies that report HNSCC incidence in non-white population, and a similar analysis will be conducted in the future to include more equally distributed races [39,40,41,42]. There was an apparent absence of HPVP HNSCC patients in the oldest patient category (76–85 years of age—Figure 1B), and we theorize that might be due to the fact that HPVP HNSCC are significantly more rare than HPVN patients, thus causing this age groups’ underrepresentation. Alternatively, the HPVP HNSCC patients do not survive for a long period to be included in the data (76–85 years of age) [43,44]. We observed differences in anatomical sites of HNSCC that were dependent on the HPV status (Figure 1E–G). Tonsil was the predominant location in HPVP patients, while the oral tongue had the most cases in HPVN patients (Figure 1F,G). In the US, regardless of HPV status, the oral tongue is the most common site for HNSCC [39].

In our analysis, genes that play a role in all HNSCC development belonged to four main functional pathways: Cell survival, cellular proliferation, squamous epithelial differentiation, and invasion/metastasis. We identified differentially expressed and methylated genes in HPVP versus HPVN HNSCC. Of the 1854 DEG, 16 genes were the top hits identified in the transcriptome and methylome analyses. The functions of these genes range from cell cycle, immune response, to cell death regulation. Specifically, we found that *SYCP2* and *TAF7L* were the two most deregulated genes in both analyses. Synaptonemal complex protein 2 (*SYCP2)* was the top hypomethylated and upregulated gene in HPVP HNSCC. This gene is the testis-specific human gene and has been associated with impaired meiosis [45]. It is known that *SYCP2* aberrant expression in HPVP cancers may contribute to the genomic instability induced by high-risk HPVs and subsequent oncogenic change [46]. In 2015, a paper by Masterson et al. reported that deregulation of *SYCP2* predicts early-stage human papillomavirus-positive oropharyngeal carcinoma. The same authors concluded their study by proposing *SYCP2* as a potential biomarker [47]. In addition, an independent study showed that *SYCP2* was hypomethylated in HPVP HNSCC, which is in concordance with what we have discovered [19]. This might imply that the previously proposed biomarker function for *SYCP2* is not unlikely. In addition to these reports, the elevated expression of *SYCP2* in HPV-associated tumors has previously been observed in three additional gene expression analysis studies [48,49,50]. The human protein atlas reports the highest expression of SYCP2 in male tissues, while this protein is also expressed in female tissues, although less (https://www.proteinatlas.org/ENSG00000196074-SYCP2/tissue, accessed on 13 September 2022). All of this suggests that SYCP2 is involved in more than its primary function as the synaptonemal complex protein when deregulated. Additional research is needed to determine the significance of SYCP2 levels in male and female samples. Similarly, the second highlighted gene that was hypomethylated and upregulated in HPVP HNSCC is TATA-box binding protein associated factor 7-like (*TAF7L*), a gene involved in spermatogenesis [51]. According to a study by Mobasheri et al., *TAF7L* is upregulated in breast cancer; therefore, it is possible that it is not an exclusive feature, which is observed only in breast cancer tissue [52].

DEG analysis identified that *PCNA*, *TNFRSF14*, *TRAF1*, *TRAF2*, *BIRC3*, and *BCL2* were significantly altered in HPVP HNSCC.

Proliferating cell nuclear antigen (*PCNA*) is a gene that was significantly overexpressed in HPVP versus HPVN HNSCC patient samples. It has been shown that *PCNA* expression levels change during cell cycle, as *PCNA* is associated with proliferation and cell transformation in cancer [53,54]. *PCNA* is one of the crucial regulators in cell cycle as it forms complexes with cell cycle activators (cyclins and cyclin dependent kinases) and inhibitors (*p21*) [53]. Post-translational modifications are crucial for the *PCNA* function, significantly, that *PCNA* exists in an alternative methylated form in cancers [55].

Tumor necrosis factor receptor superfamily member 14 (*TNFRSF14*) is known to be a herpesvirus entry mediator by being a part of signal transduction pathways that activate inflammatory and inhibitory T-cell immune response [56]. It is not surprising to observe that it was upregulated in HPVP HNSCC, although it is interesting that a herpesvirus-related gene has been upregulated upon HPV infection in this cancer type. *TNFRSF14* is known to interact with TNF receptor associated factor 2 (*TRAF2*), which is also upregulated in HPVP HNSCC. This protein directly interacts with the TNF receptors, and forms a complex with another TRAF family member, *TRAF1* which is also upregulated in HPVP HNSCC. This is all necessary for *TNFα*-mediated activation of *MAPK8/JNK* and *NF-kβ*, which are known to be involved in cell survival. The protein complex formed by *TRAF2* and *TRAF1* interacts with the inhibitor-of-apoptosis proteins (IAPs), and functions as a mediator of the anti-apoptotic and pro-survival signals from TNF receptors. One of those IAPs that is upregulated in HPVP HNSCC is *BIRC3*-apoptosis inhibitor [57,58,59]. According to The Human Protein Atlas (THPA), *TRAF2* has the highest expression in HNSCC, followed by cervical cancer among all sampled cancer types (17 cancer types) [60]. *BIRC3* shows similar observations, implying that this pattern may be specific for HPV-related HNSCC [60]. Another role of *TRAF1* is a negative regulation of Toll-like receptor (TLR) and Nod-like receptor (NLR) signaling. *TRAF1* can also, independently from *TRAF2*, contribute to *NF-kβ* activation; conversely, during TLR and NLR signaling, *TRAF1* can also negatively regulate *NF-kβ* activation. According to THPA, *TRAF1* has been found to be overexpressed in HNSCC. Additionally, *TRAF1* can contribute to chronic viral infection and limit inflammation, contributing to the survival of Epstein-Barr virus dependent cancers [57,60]. TRAF family genes (*TRAF1* and *TRAF2,* specifically) have been found to be differentially expressed in a couple of HPV-related studies, including one in our lab [61,62]. An interesting question follows: Does *TRAF1* have a similar role in HPV-dependent cancers, as well? To investigate this, more research is required.

In addition to *BIRC3*-apoptosis inhibitor which is upregulated in HPVP HNSCC, *BCL-2*, an anti-apoptotic gene has been observed to be upregulated in HPVP HNSCC, as well. An existing model explains the observed picture in our data. Similarly to oncogene addiction, some tumor cells may be dependent on *BCL-2* for survival [63]. As tumor environment may induce higher stress signal production that is pro-apoptotic in nature, a proportion of cancer cells manage to overexpress *BCL-2* and survive the production of this anti-apoptotic signal. In this way, *BCL-2* helps cancer progression by promoting the survival of altered cells [64,65]. Moreover, *BCL-2* is known to be overexpressed in non-hematologic tumors as ovarian, neuroblastoma, colorectal, and HNSCC [66,67,68,69].

Starburst analysis combined DEG and DMR results and highlighted genes that were the most hypomethylated and upregulated and the most hypermethylated and downregulated. We performed Starburst with FDR cutoff = 1 and a more stringent parameter FDR cutoff = 3 and maintained the top highlighted gene profile (Supplementary Table S4, and Figure 4C,D), specifically *SYCP2* and *TAF7L*. Considered together, some of the DEG identified as top hits may be used as potential biomarkers for early identification of HPVP HNSCC, including *SYCP2*, *TAFL7,* and *ZFR2*. The analysis of DEG of tonsil HPVP HNSCC and oral tongue HPVN HNSCC (predominant anatomical locations of samples), identified unique genes that were downregulated in HPVP tonsil HNSCC (Supplementary Table S5). Of these genes, *RBM24,* is shown to mediate repression of p53/TP53 mRNA translation and *INHBA*, a member of the transforming growth factor-beta (*TGF*-β) superfamily of proteins. According to THPA, the highest expression of *RBM24* is observed in HNSCC, followed by cervical cancer, although we have not seen its use as a diagnostic tool [60,70]. This implies that when these genes are downregulated, this might specifically indicate HPVP HNSCC site specific (tonsil) cancer development.

## 4. Methods

### 4.1. Study Design, Patient Samples, and Analysis Workflow

**Figure 5:**
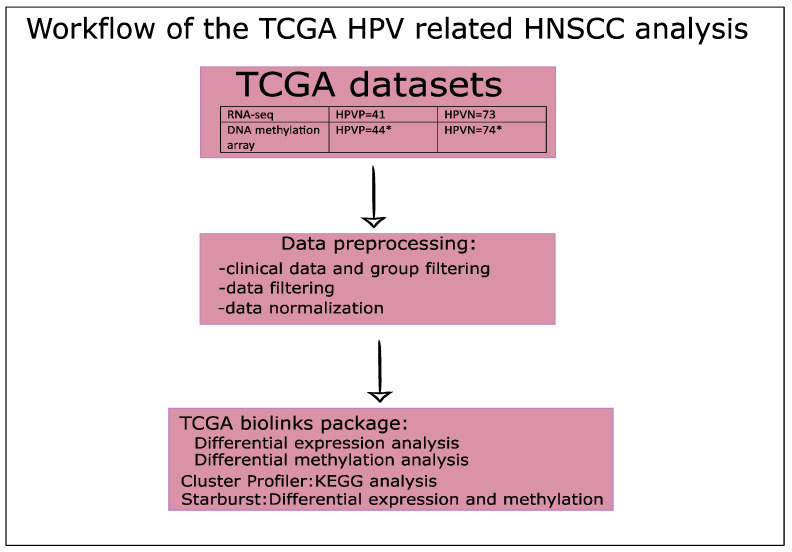
Workflow of the TCGA HPV-related HNSCC data. A schematic representation of the stepwise workflow of TCGA data analysis. * Clinical data analysis was performed only on RNA-seq patients’ data, and not on DNA methylation data.

In this study, data were acquired through the publicly available database TCGA and NCI Genomic Data Commons (GDC) [26,27]. We focused on HNSCC tumor data, and all data used in this study were open access (downloaded in 2019). We grouped the HNSCC patient samples in two experimental groups: (1) HPVP HNSCC, and (2) HPVN HNSCC. We were interested in comparing gene expression and methylation state of tumors in the absence or presence of HPV. The TCGA gene expression and DNA methylome data were extracted from RNA-seq studies of HNSCC, and from DNA methylation arrays, respectively. Moreover, we requested corresponding clinical data [27]. We used the clinical information to filter the samples into HPVP or HPVN HNSCC. We used two criteria to determine the presence of HPV: (1) The expression levels of *p16* gene, a well-known tumor-suppressor gene indicative of high-risk HPV-related cancers [71]; and (2) we used the in situ hybridization information for *p16* gene if the expression information of *p16* was not available. Using these criteria, we were able to acquire the information from 73 HPVN patients and 41 HPVP patients from the transcriptome studies, and 74 HPVN and 44 HPVP patients from the DNA methylome studies (detailed list of patients in Supplementary Table S6). For the patients that we had RNA-seq data available, we performed the analysis on clinical status, as well. To visualize clinical data, we used gplots, ggplot2, RColorBrewer, and colorRamps Bioconductor packages [72,73,74,75]. TCGA data consisted of already mapped reads that were downloaded using Bioconductor’s package TCGAbiolinks for TCGA data handling. R (version 3.6.1) and RStudio software were used for all data analyses [36,76,77,78,79,80]. Figure 5 shows our overall workflow, with each part described in detail in the following sections.

### 4.2. Data Preprocessing to Normalize Data

We preprocessed and filtered the data according to the parameters of HPV status. Preprocessing makes the data as uniform as possible, rearranges, and enables it for the analysis software to handle it. Moreover, we normalized the data to be able to perform subsequent clustering steps. Data were filtered using TCGAbiolinks and xlsx packages, and used embedded functions TCGAanalyze_Preprocessing, TCGAanalyze Normalization, and TCGAanalyze_Filtering [36,81].

### 4.3. Data Clustering Analyses

To investigate whether clustering was as expected (HPVP versus HPVN HNSCC), principal component analysis (PCA) and hierarchical clustering with heatmaps using edgeR and gplots packages, and heatmap.2 function in R were performed [75,82,83]. For the PCA analysis, we used prcomp function already existent in R, and for the hierarchical clustering with heatmaps, we used edgeR package for R and gplots, ggplot2, and RColor Brewer libraries for data visualization throughout the analyses [72,73,75,82]. For heatmap clustering, we followed a recommended online tutorial [84]. Using heatmap clustering, we investigated the most variable transcripts, as well as the genes that have the highest mean values across 114 patients, using it as a proxy for the most abundant transcripts.

### 4.4. Transcriptome Analysis: Differential Gene Expression Analysis (DGE) and Pathway Analysis

To understand differential gene expression of the filtered data, a DGE analysis was performed using TCGAbiolinks TCGAanalyze_DEA function. We used a false discovery rate (FDR) cutoff of 0.01, which represents a threshold to filter DEGs according to their corrected *p*-value. Moreover, a probe expression fold change (logFC) cutoff of 1 was used. To understand the nature of the extracted deregulated genes, we performed a pathway analysis using clusterProfiler Bioconductor package, and the function enrichKEGG, along with packages SummarizedExperiment, MultiAssayExperiment, and genefilter [85,86,87,88]. To visualize the identified pathways, we used pathview Bioconductor package, and to visualize DEG in a volcano plot we used EnhancedVolcano Bioconductor package [31,89]. We used PANTHER (Protein ANalysis THrough Evolutionary Relationships) and Enrichr, two comprehensive gene set enrichment analysis tools, to investigate the enriched pathways in the DEG dataset [32,33,34].

### 4.5. DNA Methylome Analysis: Differential Methylation Analysis (DMA)

To analyze the DNA methylation patterns, we used TCGAbiolinks function TCGAanalyze_DMR, and used *p*-value cutoff = 10^−5^ and $\bar{\beta \;}$ ≥ 0.25. “$\bar{\beta \;}$” is a parameter for differential methylation levels that ranges between 0 and 1, 0 being unmethylated and 1 being fully methylated.

### 4.6. Starburst Analysis: Integrative Analysis of DEG and Differentially Methylated Regions (DMR)

To observe common patterns of gene silencing or overexpression, we combined the two datasets (DEG and DMR) using TCGAbiolinks TCGAvisualize_starburst function [36]. We used $\bar{\beta \;}$ ≥ 0.25, FDR_expression_ ≤ 10^−5^, FDR_DNAmethylation_ ≤ 10^−5^, and │logFC│ ≥ 1. Moreover, we tested the data with a more stringent parameter of │logFC│ ≥ 3, and decided to work with the former parameter, as the analysis demonstrated that the most prominent genes were filtered under both parameters.

## 5. Conclusions

In conclusion, using TCGA transcriptome data enabled us to identify 1854 DEG, and these DEG belong to a wide range of pathways, including cell cycle, papillomavirus infection, transcriptional misregulation, TNF signaling, cytoskeletal rearrangement, and apoptosis. Combining the knowledge gained, both by transcriptome and DNA methylome data analyses, we identified potential players that might contribute to cancer development in HPVP HNSCC. In particular, *SYCP2* and *TAF7L*, which have been shown in the past to be deregulated in cancer development [46,47,52]. *SYCP2* specifically attracts our attention, as it has been shown that deregulation of *SYCP2* predicts early stage HPVP oropharyngeal carcinoma and it has been proposed to serve as a biomarker by other authors [47]. Moreover, we propose a potential panel of genes to serve for HPVP HNSCC detection and possible anatomical characterization. Screening for circulating tumor DNA from peripheral blood is low invasive and provides fast results, and we suggest screening for HPVP HNSCC using a panel, including *RBM24*, *INHBA*, *SYCP2*, *TAFL7,* and *ZFR2*. This may serve as an informative tool for HNSCC HPVP screening, and even for the detection of the specific anatomical location.

## Figures and Tables

**Table 1:** Representative enriched KEGG pathways with some of the top enriched respective genes from the DEG pool.

Representative KEGG Pathways	Mapped Differentially Expressed Genes in HPVP vs. HPVN
ECM-receptor interaction	CD36, ITGA6, ITGA5, ITGB3
Focal adhesion	BCL2, EGF, EGFR, ERBB2, IGF1, VEGFC
Viral protein interaction with cytokine and cytokine receptor	IL18, IL18RAP, IL19, LTA, TNFRSF14, IL6
Proteoglycans in cancer	TP53, EGFR, ERBB2, IGF1
Transcriptional misregulation in cancer	TP53, BCL2A1, CCNA1, CDKN2C, CSF2, GADD45G, ID2, IL6, MYCN, MEF2C, TLX3, TRAF1
Human papillomavirus infection	CCNE2, CDK6, E2F1, PDGFRB, EGF, EGFR, TP53
TNF signaling pathway	BIRC3, CCL20, CSF2, IL15, IL6, TRAF1, TRAF2, VEGFC
Cell cycle	BIRC3, BCL2, BCL2A1, NGF, TRAF1, TRAF2, PCNA, TP53, GADD45G
TGF-beta signaling pathway	AMH, DCN, ID2, IFNG, INHBA, INHBB, LTBP1, NOG, THBS1
Apoptosis	BCL2, BCL2A1, BIRC3, GADD45G, NGF, TP53, TRAF1, TRAF2

**Table 2:** Representative enriched pathways, ontologies, and transcription factors filtered by A. Enrichr and B. PANTHER (from November 2019).

Category	Regulation Level	q-Value	Database
**Transcription Factors**			
NFkB	upregulated	3.29 × 10^−2^	TRANSFAC and JASPAR PWMs
SP1 human	downregulated	2.37 × 10^−6^	TRRUST Transcription Factors 2019
**Pathways**			
Retinoblastoma gene in cancer WP2446	upregulated	4.58 × 10^−6^	WikiPathways~2019 Human
DNA strand elongation Homo Sapiens R-HAS-69190	upregulated	2.02 × 10^−4^	Reactome~2016
Beta1 integrin cell surface interactions Homo Sapiens	downregulated	1.25 × 10^−15^	NCI-Nature 2016
ITGB1	downregulated	5.64 × 10^−5^	PPI Hub Proteins
Integrin signaling pathway Homo Sapiens	downregulated	2.02 × 10^−6^	PANTHER 2016
**Ontologies**			
G1/S transition in mitotic cell cycle (GO:0000082)	upregulated	3.04 × 10^−2^	GO Biological Processes 2018
T cell receptor complex (GO:0042101)	upregulated	7.57 × 10^−5^	GO Cellular Component 2018
Collagen binding (GO:0005518)	downregulated	1.70 × 10^−8^	GO Molecular Function 2018

**Table 3:** Representative enriched pathways, ontologies, and transcription factors filtered by PANTHER (from November 2019).

Upregulated Processes	Downregulated Processes
**Category: Biological Processes**	**Category: Biological Processes**
Cellular Process was top hit (GO:0009987) 311/913 genes	Cellular Process was top hit (GO:0009987) 344/941 genes
**subcategory**	**subcategory**
cell cycle (GO:0007049) 44/311 genes	cellular response to stimulus (GO:0051716) 110/344 genes

**Table 4:** Top 9 most statistically significant KEGG pathways obtained with the DEG from the differential expression analysis.

ID	Pathway	Gene Ratio	*q*-Value
hsa04512	ECM-receptor interaction	32/714	3.47 × 10^−10^
hsa04060	Cytokine-cytokine receptor interaction	61/714	3.38 × 10^−8^
hsa04640	Hematopoetic cell lineage	30/714	5.86 × 10^−8^
hsa04974	Protein digestion and absorption	29/714	1.19 × 10^−7^
hsa04510	Focal adhesion	44/714	6.22 × 10^−7^
hsa05410	Hypertrophic cardiomyopathy (HCM)	25/714	8.20 × 10^−6^
hsa04151	PI3K-Akt signaling pathway	61/714	1.42 × 10^−5^
hsa04061	Viral protein interaction with cytokine and cytokine receptor	26/714	1.47 × 10^−5^
hsa05150	Staphylococcus aureus infection	24/714	6.90 × 10^−5^

**Table 5:** Top 5 hypo- and hypermethylated genes among HPVN and HPVP HNSCC patients. HPVN is used as a baseline.

Probe ID	Gene Symbol	Adjusted *p*-Value	Status in HPVP
cg11456145	CDC42EP5	8.40 × 10^−11^	Hypomethylated
cg07915849	ABCA17P;ABCA3	6.97 × 10^−10^	Hypomethylated
cg10504436	DERL3	6.97 × 10^−10^	Hypomethylated
cg12181372	SYCP2	1.77 × 10^−9^	Hypomethylated
cg22220310	SDF4;B3GALT6	2.08 × 10^−9^	Hypomethylated
cg07907859	FAM133A;NAP1L3	2.90 × 10^−9^	Hypermethylated
cg00757182	ZNF773	4.81 × 10^−9^	Hypermethylated
cg12387713	MSX2	5.08 × 10^−9^	Hypermethylated
cg13458645	PITX2	5.08 × 10^−9^	Hypermethylated
cg11876013	SCHIP1	5.29 × 10^−9^	Hypermethylated

**Table 6:** Representative top differentially expressed genes and differentially methylated regions filtered by Starburst (FDR cutoff = 1; HPVN group is used as a baseline).

Gene Symbol	Status in HPVP	Gene Name
TAF7L	Hypomethylated	*TATA-Box Binding Protein Associated Factor 7 Like*
SYCP2	Hypomethylated	*Synaptonemal Complex Protein 2*
LOC285954;INHBA	Hypermethylated	*Inhibin Subunit Beta A*
SULF1	Hypermethylated	*Sulfatase 1*
CCNA1	Hypermethylated	*Cyclin A1*

## Data Availability

Data used in this study are open access and can be found on NIH GDC Legacy Archive under the following link: https://portal.gdc.cancer.gov/legacy-archive/search/f, accessed on 30 August 2019.

## Supplementary Materials

The following supporting information can be downloaded at: https://www.mdpi.com/article/10.3390/ijms231810967/s1.

## Abbreviations

HPV	Human Papillomavirus
HNSCC	head and neck squamous cell carcinoma
pRb	Retinoblastoma protein
PCNA	proliferating cell nuclear antigen
TNFRSF14	Tumor Necrosis Factor (TNF) Receptor Superfamily Member 14
TRAF1	TNF Receptor Associated Factor 1
TRAF2	TNF Receptor Associated Factor 2
BCL2	B-cell lymphoma 2 apoptosis regulator
BIRC3	Baculoviral Inhibitor of Apoptosis (IAP) Repeat Containing 3
SYCP2	Synaptonemal Complex Protein 2
TAF7L	TATA-Box Binding Protein Associated Factor 7-Like
E6	HPV early gene 6
E7	HPV early gene 7
TP53/p53	tumor suppressor gene/protein p53
HPVN	HPV negative HNSCC
HPVP	HPV positive HNSCC
p16	cyclin-dependent kinase inhibitor
CCND1	cyclin D1
TCGA	The Cancer Genome Atlas
GDC	NCI Genomic Data Commons
PCA	Principal Component Analysis
DGE	differential gene expression
DMA	differential methylation analysis
DMR	differential methylated regions
GPX2	Glutathione peroxidase 2
KRTDAP	Keratinocyte Differentiation Associated Protein
CRNN	Cornulin
PCA	Principal component analysis
KEGG	Kyoto Encyclopedia of Genes and Genomes
ECM	extracellular matrix
PANTHER	protein annotation through evolutionary relationship
THPA	The Human Protein Atlas
TLR	Toll-like receptor
NLR	Nod-like receptor
NF-kβ	nuclear factor kappa-light-chain-enhancer of activated B cells
ZFR2	Zinc Finger RNA Binding Protein 2
INHBA	Inhibin Subunit Beta A
